# Systematic Review on Local Ancestor Inference From a Mathematical and Algorithmic Perspective

**DOI:** 10.3389/fgene.2021.639877

**Published:** 2021-05-24

**Authors:** Jie Wu, Yangxiu Liu, Yiqiang Zhao

**Affiliations:** ^1^State Key Laboratory of Agrobiotechnology, China Agricultural University, Beijing, China; ^2^Institute of Chinese Materia Medica, China Academy of Chinese Medical Sciences, Beijing, China

**Keywords:** LAI model, HMM, mathematical structure, bibliometrics, benchmark

## Abstract

Genotypic data provide deep insights into the population history and medical genetics. The local ancestry inference (LAI) (also termed local ancestry deconvolution) method uses the hidden Markov model (HMM) to solve the mathematical problem of ancestry reconstruction based on genomic data. HMM is combined with other statistical models and machine learning techniques for particular genetic tasks in a series of computer tools. In this article, we surveyed the mathematical structure, application characteristics, historical development, and benchmark analysis of the LAI method in detail, which will help researchers better understand and further develop LAI methods. Firstly, we extensively explore the mathematical structure of each model and its characteristic applications. Next, we use bibliometrics to show detailed model application fields and list articles to elaborate on the historical development. LAI publications had experienced a peak period during 2006–2016 and had kept on moving in the following years. The efficiency, accuracy, and stability of the existing models were evaluated by the benchmark. We find that phased data had higher accuracy in comparison with unphased data. We summarize these models with their distinct advantages and disadvantages. The Loter model uses dynamic programming to obtain a globally optimal solution with its parameter-free advantage. Aligned bases can be used directly in the Seqmix model if the genotype is hard to call. This research may help model developers to realize current challenges, develop more advanced models, and enable scholars to select appropriate models according to given populations and datasets.

## Introduction

Rapid advancements in computing technologies, genome sequencing, and single nucleotide polymorphism (SNP) genotyping methods have made it possible to infer the genomic structure at a fine scale (Kidd et al., 2012). It also accelerates the exploration of mixed ancestry or local ancestry inference (LAI) at the individual and population levels (Schumer et al., 2020). In LAI, each chromosome is considered as a mosaic of genomic segments, originated from multiple ancestral groups (Padhukasahasram, 2014). LAI is of great importance in studying population evolution, migration history, or disease risks (Fitak et al., 2018). Up to now, various LAIs have been widely used; each model comes with its own advantages and disadvantages toward LAI in admixed populations (Geza et al., 2019).

Due to the genetic recombination after interbreeding, the genome consists of mosaic of DNA segments with different genetic ancestries (Dougherty et al., 2017). Genotypes from putative ancestral populations are mostly utilized to infer the local ancestry of admixed individuals (Sankararaman et al., 2008a). Currently, about 70% of LAI models are based on hidden Markov model (HMM), where the hidden states correspond to ancestries and generate the observed haplotypes/genotypes (Baran et al., 2012). LAI models use ancestry informative markers (AIMs) for simplicity or to account for linkage disequilibrium (LD) of variants, i.e., STRUCTURE (Falush et al., 2003), Hapmix (Price et al., 2009), Saber (Tang et al., 2006), and LAMP-LD (Baran et al., 2012). Other models consider rich haplotype information by employing window-based strategies, i.e., RFMix (Maples et al., 2013), PCAdmix (Brisbin et al., 2012), and LAMP (Sankararaman et al., 2008b). Table 1 presents more details in this regard.

**TABLE 1:** Detailed deconvolution model description.

Model name	Split format	Algorithm	The number of ancestral populations	Built-in phasing error correction	Reference populations	Admixed populations	LD	References
Hapmix	Haplotype	HMM	2	Yes	Phased	Unphased	Yes	Price et al., 2009
Seqmix	Haplotype	HMM	2	No	Unphased	Unphased	No	Hu et al., 2013
PCAdmix	Window	PCA + HMM	> = 2	No	Phased	Phased	Yes	Brisbin et al., 2012
Supportmix	Window	SVM +HMM	> = 2	No	Phased	Phased	Yes	Omberg et al., 2012
LAMP-LD	Window	HMM	2, 3, 5	No	Phased	Unphased	Yes	Baran et al., 2012
ALLOY	Window	F-HMM	> = 2	Yes	Phased	Phased	Yes	Rodriguez et al., 2013
Saber	Haplotype	M-HMM	> = 2	No	Phased/Unphased	Phased/Unphased	Yes	Tang et al., 2006
SWITCH	Haplotype	MCMC	> = 2	No	Phased	Phased	Yes	Sankararaman et al., 2008a
HAPAA	Haplotype	H-HMM	> = 2	Yes	Phased	Phased	Yes	Sundquist et al., 2008
ELAI	Haplotype	Two-layer HMM	> = 2	No	Phased/Unphased	Phased/Unphased	Yes	Guan, 2014
RFMix	Window	RF + CRF	> = 2	No	Phased	Phased	No	Maples et al., 2013
Chromopainter	Haplotype	PCA+MCMC	> = 2	No	Phased	Phased	Yes	Lawson et al., 2012
LAMP	Window	ICM	> = 2	No	Unphased	Unphased	No	Sankararaman et al., 2008b
Loter	Haplotype	DP	> = 2	Yes	Phased	Phased	No	Dias-Alves et al., 2018
EILA	Haplotype	FQR + *K*-means	> = 2	No	Unphased	Unphased	No	Yang et al., 2013
LASER 2.0	Haplotype	PCA + PPA	> = 2	No	Phased	Phased	No	Wang et al., 2015
WINPOP	Window	DP	> = 2	Yes	Unphased	Unphased	No	Pasaniuc et al., 2009

## Models Based on an Original Hidden Markov Model

The challenge of identifying ancestry along each chromosome can be addressed with different approaches. One of the most widely used models is HMM, an extension of a Markov chain, in which the state transformation is generally unobservable (Wu and Zhao, 2019). In HMM, the parameters include initial state distributions, state transition probability matrix, and emission probability matrix. Algorithms were developed to solve three main questions of HMM: evaluation (forward algorithm), decoding (Viterbi algorithm), and training (Baum–Welch algorithm including expectation maximization or maximum likelihood) (Schuster-Böckler and Bateman, 2007).

LAI models based on the original HMM algorithm include Hapmix (Price et al., 2009), Seqmix (Hu et al., 2013), PCAdmix (Brisbin et al., 2012), Supportmix (Omberg et al., 2012), and LAMP-LD (Baran et al., 2012). These models use Baum–Welch to iteratively update the initialized transition probability matrix and the emission probability matrix and use Viterbi for estimating the hidden ancestral states. The designs of the initialized emission and the subsequent calculations mainly differentiate among the models. Supportmix utilizes a support vector machine (SVM) (Haasl et al., 2013) for classifying the chromosome segments of the ancestral group, while PCAdmix calculates Euclidean distances between the ancestral groups and admixed individuals for finding the closest ancestry for each window.

### Hapmix Model

The Hapmix model (Price et al., 2009) is based on a combination of the HMM and haplotype. The hidden state for position *s* is denoted *via* a triplet (*i*,*j*,*k*); here, *i* denotes the ancestry derived from a different population, while *j* recorded the population from which the haplotype was copied considering miscopying, and *k* corresponds to the source of the individual the chromosomal segment was copied from. *p*^*s*^(*i*,*j*,*k*;*l*,*m*,*n*) is the transition probability from state (*i*,*j*,*k*) to state (*l*,*m*,*n*) between the adjacent sites *s* and (*s* + 1). *e*^1^*_*ijk*_*(*s*) denotes the type 1 offspring chromosome probability at site *s* and *t*_*jk*_ represents the parent individual *k* type in the reference population *j*. The initialized emission probability matrix is given in Equation (1).

(1) $$e_{i\,j\,k}^{1}\,\left(s\right)=\left\{ \begin{array}{c} \theta_{i}\delta \left(t_{j\,k}=0\right)+\left(1-\theta_{i}\right)\delta \left(t_{j\,k}=1\right)ifi=j\\ \theta_{3}\delta \left(t_{j\,k}=0\right)+\left(1-\theta_{3}\right)\delta \left(t_{j\,k}=1\right)ifi\neq j \end{array}\right.$$

Here, offspring carrying the identical type to the specific parent is with a probability (1 – θ_1_), while a different type with the probability θ_1_, θ_3_ denotes the mutation rate in the case that offspring copied from the other population.

### Seqmix Model

The Seqmix model (Hu et al., 2013) aligns bases directly rather than relying on genotypic calls. The method implemented in Seqmix consists of three layers: the hidden ancestry state, the hidden genotype, and the observed sequence reads. The genotype is placed in the intermediate layer by connecting the sequence reads and ancestry. In the HMM, the transition matrix denotes the hidden ancestry state *q*_*s*_ as (*A*_*s*__1_, *A*_*s*__2_), Herein, *A*_*s*__1_ represents the first chromosome ancestry at site *s*, while the ancestry of the other chromosomes is represented by *A*_*s*__2_. *γ_*s,s*_*_+ 1_ is the rate of recombination per generation between site *s* and *s* + 1 and *T* represents the generations since admixture. *π_*A*_* and *π_*E*_* correspond to the prior probabilities for populations 1 and 2. The initialized transition probability matrix is given in Equation (2).

(2) Ps,s+1=[Ps,s+1E,E⁢Ps,s+1E,APs,s+1A,E⁢Ps,s+1A,A]=[1-(1-e-γs,s+1⁢T)⁢πA⁢(1-e-γs,s+1⁢T)⁢πA(1-e-γs,s+1⁢T)⁢πE⁢ 1-(1-e-γs,s+1⁢T)⁢πE]

The initialized emission probability is *P*(*O_*s*_| q_*s*_*), which is calculated as a sum of the overall possible genotypes, assuming the Hardy–Weinberg equilibrium, and is weighted by ancestry-specific allele frequencies: *P*(*O*_*s*_|*q*_*s*_ = (*A*_*s*1_,*A*_*s*2_)). The genotype likelihood *P*(*O_*s*_| q_*s*_*) is the probability of the observed set of reads given the hidden ancestry state.

### PCAdmix Model

The PCAdmix model (Brisbin et al., 2012) is based on a combination of the HMM and principal component analysis (PCA). The principal components (PCs) of the ancestral populations are firstly calculated based on the phased genotypes of the ancestral representatives and the phased genotypes of admixed individuals projected onto the component space. The vector *P*(*S*_*i*,*w*_|*anc*_*i*,*w*_ = *j*) defines the emissions probability, anc*_*i,w*_* denotes the ancestry of haplotype *i* at window *w* from population *j* and comprises the ancestry scores across the first *K* – 1 PCs, where *K* is the total count of ancestral populations, the weighted sum *S*_*iw*_ = *L_*w*_g_*iw*_* is the ancestry score for haplotype *i* in window *w*, *g*_*iw*_ represents a column vector of the haplotype’s alleles in the window, and *L*_*w*_ represents a matrix in which the individual columns carry the PC loadings of one SNP in the window; each window is used as the observation value in HMM. The transition probability is *P*(anc*_*i,w*_* = *j|* anc*_*i,w*_*_–_ *_1_* = *k*). A forward–backward algorithm is applied to find the posterior probability for each window in the admixed haplotype.

### Supportmix Model

In the Supportmix model (Omberg et al., 2012), SVM and HMM algorithms are combined, and independent SVM classifiers are firstly applied for each genomic window to retrieve putative ancestry origins. The outputs of the SVMs are then fed to HMM to refine the ancestral assignment for each window. The emission possibilities are *p* for the hidden state (1 – *p*)/(*k*′ – 1) and for the other states, where *k*′ is the number of ancestral populations and *p* is the classification from the SVM at the corresponding window. LD is considered in the HMM where the recombination is modeled as a Poisson process. The transition probability is thus defined as (1 – *e*^–^*^*gd*^*)/(*k*′ – 1), where *d* is the genetic distance (in centimorgan) between the windows and *g* is the generation since admixture.

### LAMP-LD Model

The LAMP-LD model (Baran et al., 2012) uses a window-based HMM, which divides the genome into non-overlapping windows of fixed length *L* with a fixed state space of hidden ancestry of $\left(\begin{array}{c} K\\ 2 \end{array}\right)$. The admixed chromosome is modeled by HMM corresponding to each ancestry pair $S^{w}=\left(M_{1}^{w},M_{2}^{w}\right)$. Genotypic block *G*^*w*^ is emitted by each state$\left(M_{1}^{w},M_{2}^{w}\right)$ with the emission probability: $\sum_{\left(H_{1}^{w},H_{2}^{w}\right)}P\,\left(H_{1}^{w}|M_{1}^{w}\right)\,\left(H_{2}^{w}|M_{2}^{w}\right)$. Here, *P*($H_{1}^{w}|M_{1}^{w}$) is the probability that the haplotype segment $H_{1}^{w}$ is emitted under the ancestry *M*_1_ and $\left(H_{1}^{w},H_{2}^{w}\right)$ is the haplotype pair consistent with the genotypes. The transition probability between the two states in a consecutive window $\left(M_{1}^{w},M_{2}^{w}\right)$ and $\left(M_{1}^{w^{\prime }},M_{2}^{w^{\prime }}\right)\,$ is set to the average recombination rate per base per generation θ = 10^–8^ × *D* (*D* denotes the length in base pairs between windows) if the unordered ancestry pairs $\left(M_{1}^{w},M_{2}^{w}\right)$ and $\left(M_{1}^{w^{\prime }},M_{2}^{w^{\prime }}\right)$ differ by one ancestry, θ^2^ if both ancestries differ, or 1 *–* 2θ – θ^2^ if there is no ancestry switch.

## Models Based on a Hidden Markov Model Family

The HMM family, based on an extension of the original algorithm, includes factorial-HMM (F-HMM), hierarchical-HMM (H-HMM), Markov-HMM (M-HMM), conditional random field (CRF), and two-layer HMM. Their transition and emission probabilities have been improved for reinforcing the learning of the original HMM. LAI models based on the HMM family include ALLOY (Rodriguez et al., 2013), Saber (Omberg et al., 2012), HAPAA (Sundquist et al., 2008), ELAI (Guan, 2014), and SWITCH (Sankararaman et al., 2008a). ALLOY applies a F-HMM to get hold of the parallel process, thus giving rise to the paternal and maternal admixed haplotypes. This, in turn, strengthens the correction of the HMM parameters, especially for the emission probabilities. Saber and SWITCH improve and enhance the traditional emission probabilities at a marker by using the joint distribution of alleles at two neighboring markers. SWITCH depends on pairwise SNP allele frequencies between consecutive markers, whereas the Saber model relies on the allele frequencies at the two consecutive markers. Unlike the M-HMM emission probability models of SWITCH and Saber, HAPAA has an emission probability of a 5 × 5 stochastic matrix and is historically the first model of the series (Sundquist et al., 2008). Most of the transition probabilities still consider the genetic distance and generations in extended HMM. Like Supportmix, RFMix adopts a kind of multi-classification models for investigating chromosome segments of similar ancestry and uses CRF to smooth ancestral window information.

### ALLOY Model

The ALLOY model (Rodriguez et al., 2013) uses F-HMM and is an improved form of HMM to capture parallel processes for producing the maternal (*m*) and paternal (*p*) admixed haplotypes. This model is denoted by $H_{l}^{m},H_{l}^{p}$, the haplotype cluster membership drawn from *a*_*l*_ *∈ A*_*l*_ on the haplotypes at position *l*. *G*_*l*_ *∈* {*0*,1,2}, which is the observed genotype at the same marker position, represents the count of the minor allele. Across all the positions of the *L* marker, the presence of vectors of haplotype cluster memberships and genotypes are represented by$H^{\left\{m,p\right\}}=(H_{1}^{\left\{m,p\right\}},H_{2}^{\left\{m,p\right\}},\ldots ,H_{L}^{\left\{m,p\right\}}$) and *G* = (*G*_1_,*G*_2_,…,*G*_*L*_), correspondingly. In the model, the posterior marginal is first computed to infer the emission probability, given the sample of genotypes$P\,\left(H_{l}^{m},H_{l}^{p}|G\right)$ by applying the forward–backward algorithm. Local observation is made from the multiplication of the emission probability$P\left(G_{l}|H_{l}^{m}=a_{l},H_{l}^{p}=a_{l}^{\prime }\right)$ and by incorporating the transition probability of (*H*_*l*_|*H*_*l*−1_).

### Saber Model

The Saber model (Tang et al., 2006) computes the posterior probability of the hidden states in the M-HMM based on forward and backward algorithms and adds the relationship between the observed genotype along each chromosome. The transition probabilities of the initial state are given in Equation (3).

(3) $$P\left(Z_{1}=i|\pi \right)=\pi_{i},\left(i=1,\cdots ,N\right),A_{i\,j}^{\text{struct}}\left(t\right)=P\left(Z_{t}=j|Z_{t-1}=i,\tau ,\pi \right)$$

where *Z*_*t*_ represents unobserved ancestry, *π* represents the genome-wide average individual admixture, and *τ* is the time since admixing.

The distribution of *O_*t*_^*f*^* given *Z_*t*_^*f*^* is described by the emission probability; *O_*t*_^*f*^* represents the observed genotype. The allele frequency in each ancestral population is considered as a natural choice of emission probabilities at a particular marker. In M-HMM, the model further requires the alleles’ joint distribution at two neighboring markers. Equation (4) can be defined as the emission probability at marker *t.*

(4) $$B_{t}\left(v,u,j,i\right)=P\left(O_{t}^{f}=v|O_{t-1}^{f}=u,Z_{t}^{f}=j,Z_{t-1}^{f}=i\right)$$

### SWITCH Model

The SWITCH model (Sankararaman et al., 2008a) uses M-HMM and presents an effective initialization procedure that yields a highly accurate outcome at a notably reduced cost of computation *via* the expectation maximization (EM) algorithm for the estimation of parameters. In each EM iteration, the ancestry information of each haplotype is represented by matrix *Z*, and matrix *W* denotes recombination events. The *Z* and *W* updates are computed with the help of the Viterbi algorithm having emission probabilities *P*_*r*_(*X*_*i*,*j*_|*Z*_*i*,*j*_,*p*_*j*_,*q*_*j*_), which are replaced with an integral of *p*_*j*_ and *q*_*j*_; the noticed SNP binary matrix has been represented by *X*_*i,j*_ at the *j-*th SNP of the *i-*th haplotype. The expectation step includes the calculation of the posterior probabilities of *p*_*j*_ and *q*_*j*_; that is, $P_{r}\,\left(p_{j},q_{j}|X_{i,j,}\,Z_{i,j}^{\left(t\right)}\right)$. The underlined step can be performed *via* Bayes’ theorem. The maximization step includes finding a solution to *m* separate optimization problems in *Z*_*i*_, *W*_*i*_, *i*∈{1,*m*}, where the vector of ancestries for the *i-*th haplotype is represented by *Z*_*i*_ and the complementary vector of recombination events is shown by *W*_*i*_, as shown in Equation (5).

(5) $$\log\left[\mathrm{Pr}\left(Z_{i,1}\text{|}\alpha \right)\right]+I_{1,i}\left(Z_{i,1}\right)+\sum {}_{j=2}^{n}\text{\{}I_{j,i}(Z_{i,j})+f_{i,j-1,j}\left(Z_{i,j-1},Z_{i,j},W_{i,j}\right)\text{\}}.$$

where *f*_*i*,*j*−1,*j*_(*Z*_*i*,*j*−1_,*Z*_*i*,*j*_,*W*_*i*,*j*_). corresponds to the log transition probabilities and *I*_*j*,*i*_(*Z*_*i*,*j*_) represents the expectations of the log emission probabilities. α refers to the fraction of the first population in the ancestral population.

### HAPAA Model

In the HAPAA model (Sundquist et al., 2008) based on H-HMM, an integration of the model with multiple HMMs is used. The model assumes the *N* populations *P* = *{P*_1_, *P*_2_,…, *P_*N*_}*, each *P* denoted *via* a set of *n*_*p*_ model individuals, *P*_*p*_ = {*a*_*p*1_,*a*_*p*2_,…,*a*_*pn*_*p*__}. The probability of emission is given by a 5 × 5 stochastic matrix, $P\left({\bar{a}}_{i}=x|y_{i}=S_{p\,k\,h}\right)$, where the hidden state variable is denoted *y*_*i*_. *S*_*pkh*_ is for the two haplotypes *h* ∈ {0, 1} of each *k* individual in the *p* population. After that, an emitting state starts with an equivalent probability for the individual population, which is provided as *P*(*y*_1_ = *S*_*pkh*_) = 1/2*Nn*_*p*_. Every *S*_*pkh*_ state can exist in three transitions: back to itself and the other presumed haplotype in the very individual *S*_*pk* (1 –_ *_*h*_*_)_ with a probability of (1−*w*_*pki*_)*e*^−τ_*p*_*R*_*i*_^, and *w*_*pki*_⋅*e*^−τ_*p*_*R*_*i*_^, respectively, or to the state Out*_*p*_* exit with probability 1−*e*^−τ_*p*_*R*_*i*_^. Training samples provide the recombination rate *τ_*p*_*, the probability of a phasing switch error is represented by *w*_*pki*_, *R*_*i*_ represents the genetic distance between the loci, the emission probability is represented by $P\left({\bar{a}}_{i}=x|y_{i}=S_{p\,k\,h}\right)$, and the transition probability is represented by$P\left({\text{Out}}_{p}\to In_{p^{\prime }}\right)$, and using an EM algorithm to update these parameters on the training examples.

### ELAI Model

In the ELAI model (Guan, 2014), a two-layer HMM is used: the upper-layer switch probabilities provide the information regarding the switching frequency between various ancestral populations, while the lower-layer switch probabilities are related to the switching frequency between the haplotypes within each ancestral population. For each individual *i*, let *X*_*m*_^(^*^*i*^*^)^, *Y*_*m*_^(^*^*i*^*^)^ be the hidden state of the upper and lower clusters at marker *m*. Herein, *X*_*m*_^(^*^*i*^*^)^ obtains values in 1,⋯*S*, *S* and *Y*_*m*_^(^*^*i*^*^)^ obtain values in 1,⋯*K*, *K*. The haplotypic marker *h*_*m*_^(^*^*i*^*^)^ emission of *i* at *m* from a lower-layer cluster is given in Equation (6).

(6) $$P\,\left(h_{m}^{\left(i\right)}|X_{m}^{\left(i\right)},Y_{m}^{\left(i\right)},\xi \right)=P\,\left(h_{m}^{\left(i\right)}|Y_{m}^{\left(i\right)},\xi \right)$$

The complete data likelihood combines with the lower-layer and upper-layer clusters, as shown in Equation (7).

(7) $$P\left(h^{\left(1\right)},\ldots ,h^{\left(N\right)},X^{\left(1\right)},Y^{\left(1\right)},\ldots ,X^{\left(N\right)},Y^{\left(N\right)}\text{|}\xi \right)=\prod_{i\;=\;1}^{N}\;\prod_{m\;=\;1}^{M}\;P(h_{m}^{\left(i\right)}|Y_{m}^{\left(i\right)},\xi )P(X_{m}^{\left(i\right)},Y_{m}^{\left(i\right)}|\xi )$$

where *ξ* is defined as the parameter correlating with the HMM.

The first marker and the Markov transitions are expressed as follows because the model takes two scales of LD occurring in admixed individuals into consideration: *P*(*X*_1_ = s,*Y*_1_ = k) = *P*(*Y*_1_ = *k*|*X*_1_ = *s*)*P*(*X*_1_ = *s*) and *P*(*X*_*m*_ = *s*,*Y*_*m*_ = *k*|*X*_*m*−1_ = *s*′,*Y*_*m*−1_ = *k*′).

### RFMix Model

In this model (Maples et al., 2013), CRF and the random forest (RF) (Wu and Zhao, 2019) algorithm are combined. In the event of CRF along with its chain structures, all potential functions work on pairs of haplotype label variables, *H*_*i*_ and *H*_*i*_ *_+ 1_*, that are adjacent to each other. Firstly, the emission probability is learned and RF is trained with segments (reference haplotypes) in the corresponding window, which is then used for the estimation of the ancestry *A*_i,*_ posterior probabilities, considering the segment of the admixed haplotype for the window. Secondly, the transition probability is also learned. In adjacent windows, the joint probability of the local ancestries relies primarily on the global proportion of the individual ancestry and the likeliness of recombination between the pair of windows. The joint probability distribution is *P*(*A*_*i*,*p*_ = *j*,*A*_*i*,*p* + 1_ = *k*). Thirdly, a linear-chain CRF is independently used to model *P*(*A*_*i*_,_*_| H_*i*_,_*_:Θ) for each admixed chromosome. The EM method is used for updating the above parameters. In consideration of a phasing error, *P*(*A*_*i*_,_*_,*A*_*ic*_,_*_,*H*_*i*_,_*_,*H*_*ic*_,_*_| O_*i*_,_*_,*O*_*ic*_,_*_:Θ) is modeled, wherein *i* and *i*_*c*_ are the indices representing both copies of the chromosome under evaluation for a specific admixed subject, *O*_*i*_,_*_ represents the phased sequence observed for chromosome *i* given by phasing algorithms, while *H*_i,*_ indicates the set of each potential haplotype in the window.

## Models Based on Non-Hidden Markov Model Family

Along with the HMM family models, there are also some other non-HMM family models that are based on the basic algorithm and data mining techniques. For example, Loter is a parameter-free model that uses dynamic programming (DP) to obtain a globally optimal solution. Chromopainter adopts PCA for investigating chromosome segments of similar ancestry and uses Markov chain Monte Carlo (MCMC) (Gilks, 1999) to smooth ancestral segment information.

### Chromopainter Model

The Chromopainter model (Lawson et al., 2012) works based on PCA and MCMC (Gilks, 1999). Firstly, PCA uses the co-ancestry matrix *x*_*ij.*_ For each element in the matrix, *x*_*ij*_ is an estimate of the number of discrete segments of individual *i*, which is strongly correlated with the individual *j* corresponding part. The Chromopainter model is built on the assumption that the chunks $P_{q_{i}\,q_{j}}/{\overset{}{n}}_{q_{j}}$in various individuals are independent; hence, the cross individuals are multiplied, which results in a complete likelihood, as shown in Equation (8).

(8) $$F\left(x\text{|}p,q\right)=\prod_{i\;=\;1,\;j\;=\;1}^{N}\;{\left(\frac{P_{q_{i}q_{j}}}{{\hat{n}}_{qj}}\right)}^{x_{ij}/c}$$

where *c* could be considered as describing an effective number of chunks, *N* represents the number of individuals, while the individuals are represented by *j* and *i* in populations *q*_*j*_ and *q*_*i*_, accordingly. Probably a single chunk delivered from the *j* to the *i* individual is $P_{q_{i}\,q_{j}}/{\hat{n}}_{q_{j}}$, and in various individuals, the chunks are independent.

Secondly, a prior value *P*_*a*_ ∼ Dirichlet(β*_*a*_ = {β_*a1*_*, …, β*_*aK*_}*) is selected. β*_*ab*_* values are proportionate to the *a priori* estimated value of each *P*_*ab*_. Eventually, *F* is updated within the algorithm *via* the updates of standard Metropolis–Hastings MCMC.

### LAMP Model

In this model (Sankararaman et al., 2008b), a clustering algorithm called iterated conditional model (ICM) is used to investigate an optimal classification of all individuals regarding probability. The ICM algorithm is different from the traditional EM model. The *E* step comprises the expected classification θ, given minor allele frequencies *f*_*l*_, thus resulting in a fractional class membership for each individual *i*. In the LAMP, it is supposed that a logical answer will be provided by the initial classification, and it determines the maximum *a posteriori* estimate of θ, as indicated here.

For populations *A*_*s*_ and *A*_*t*_, the underlined model uses *G*_*i*_, which represents the genotype (*g*_*i1*_, …, *g*_*in*_) of the individual *i*, as shown in Equation (9).

(9) $$\hat{\theta }\left(i\right)=arg{\text{max}}_{A_{s}\,A_{t\in \left\{1,\ldots ,K\right\}}^{2}}P_{r}\left[\theta \left(i\right)=A_{s}A_{t}|f_{1},\ldots ,f_{k},G_{i}\right]$$

In the *M* step, it receives the maximum–likelihood estimation of *f*_1_,…,*f*_*k*_*via* investigation, as shown in Equation (10).

(10) $$arg{\text{max}}_{f_{1},\ldots ,f_{k},}P_{r}\left[{\left(G_{i}\right)}_{i\;\;=1}^{m}\text{|}f_{1},\ldots f_{k},\theta \right]=\prod_{i\;=\;1}^{m}P_{r}[G_{i}|f_{1},\ldots ,f_{k},\theta (i)]$$

### Loter Model

The Loter model (Dias-Alves et al., 2018) adopts DP and supposes that ancestral populations contain individuals *n*, which results in haplotypes (2*n*) presented *via* (*H*_1_, *…*, *H*_2_*_*n*_*). The *i-*th haplotype value (0 or 1) at the *j-*th SNP is indicated *via H_*i*_^*j*^*. The estimation of the haplotype *h* (admixed individual) is made possible by a vector (*s*_1_, *…*, *s*_*p*_) that determines the sequence (haplotype labels). For the *j-*th SNP in the dataset, *s*_*j*_ = *k* if haplotype *h* resulted from the haplotype *H*_*k*_ copy. The optimization problem comprised reducing the underlined cost function, as shown in Equation (11).

(11) $$C\left(s_{1},\ldots ,s_{p}\right)=\sum_{j\;=1}^{p-1}\text{|}h^{j}-H_{s_{j}}^{j}\text{|}\;+\lambda \;\sum_{j\;=1}^{p-1}1_{s_{j\neq }s_{j+1}}$$

In consideration of a phasing error, shown in Equation (12)

(12) $$C^{\prime }(\text{Θ})=\sum {}_{j\;=1}^{p}\text{|}a^{j}-A_{s_{j}}^{j}\text{|}+\sum {}_{j\;=1}^{p}\text{|}a\prime^{j}-A_{s_{j}}^{j}\text{|}+\lambda \sum {}_{j\;=1}^{p-1}1_{s_{j\neq }s_{j+1}}+\lambda \sum {}_{j\;=1}^{p-1}1_{s_{j}^{\prime }\neq s_{j+1}^{\prime }}$$

where (*s*_1_, …, *s*_*p*_) is in *{*1, …, 2*n}^*p*^*. A regularization parameter, called *λ*, is involved in an optimization problem. A high *λ* strongly penalizes the transition between the parental haplotypes of long chunks of the constant local ancestry. *A*_1_ = (0,…,0) and *A*_2_ = (1,…,1) represent two possibility ancestry states; haploid local ancestry is represented two by vectors, *a* ∈ {0,1}^*P*^ and *a*′ ∈ {0,1}^*P*^.

### EILA Model

In the EILA model (Yang et al., 2013), fused quantile regression (FQR) and the *k*-means classifier are used and are based on three steps. Firstly, EILA defines a score *e*_*j*_,*_*i*_* (a continuous variable with a range of 0–1) for the admixed genotype *g*_*j*_,*_*i*_* (= 0,1,2) as the probability that *g*_*j*_,*_*i*_* is the descendant of ancestry *A.* This is shown in Equation (13).

(13) $$e_{j,i}=P_{r}\left[g_{j,i}\in A|g_{j,1}^{\left(A\right)},\ldots ,g_{j,n_{1}}^{\left(A\right)}\text{and}g_{j,1}^{\left(B\right)},\ldots ,g_{j,n_{2}}^{\left(B\right)}\right]$$

Secondly, θ*_*j*_*,*_*i*_* is defined as a smooth series and infers the site of breakpoints for ancestral blocks by using FQR and θ*_*j*_*,*_*i*_* is estimated *via* investigating the value that minimizes $\sum_{j=1}^{m}\left|e_{j,i}-\theta_{j,i}\right|+\lambda \,\sum_{j=2}^{m}\left|\theta_{j,i}-\theta_{j-1,i}\right|$. Smaller *λ* will lead to the lowering of penalty effects. The fitted value of θ*_*j*_*,*_*i*_* is closer to the observed *e*_*j*_,*_*i*_*. Thirdly, the breakpoints for all admixed individuals are investigated, and the model infers the local ancestry for all segments between breakpoints *via k-*means to obtain a high power of inference.

### LASER 2.0 Model

In the LASER 2.0 model (Wang et al., 2015), PCA and projection Procrustes analysis (PPA) are combined. Firstly, PCA is conducted on the genotypes of a set that has been chosen from the *N* reference individuals and results in the construction of a *K*-dimensional ancestry map. For all the evaluated samples, further PCA is carried out on genotypes through overlapping markers between the *N* reference individuals and the evaluated sample and for obtaining a *K*′-dimensional map corresponding to *N* + 1 individuals (*K*′ greater than or equal to *K*). Furthermore, PPA is performed to determine the transformation optimal set on the PCA map (sample-specific) for the maximization of its resemblance with the reference ancestry map. For the similar *N* reference individuals, the two sets of coordinates are given, i.e., *X_*N*_*_×_ *_*K*_*_′_ and *Y_*N*_*_×_ *_*K*_*, and the PPA investigates a set of transformations *f* to project *X* from a *K*′-dimensional space to a *K*-dimensional space and reduces the squared Euclidean distances being added between *f*(*X*) and *Y*. Supposing that *X*, as well as *Y*, has been centered toward the origin, the objective of the model is to investigate an isotropic scaling factor, *ρ*, in such a way that the minimization of *| | ρXA* – *Y| | _*F*_^2^* and the orthonormal projection matrix *A*_*K*__′_ *_× *K*_* takes place.

### Statistics and Comparison

**FIGURE 1:**
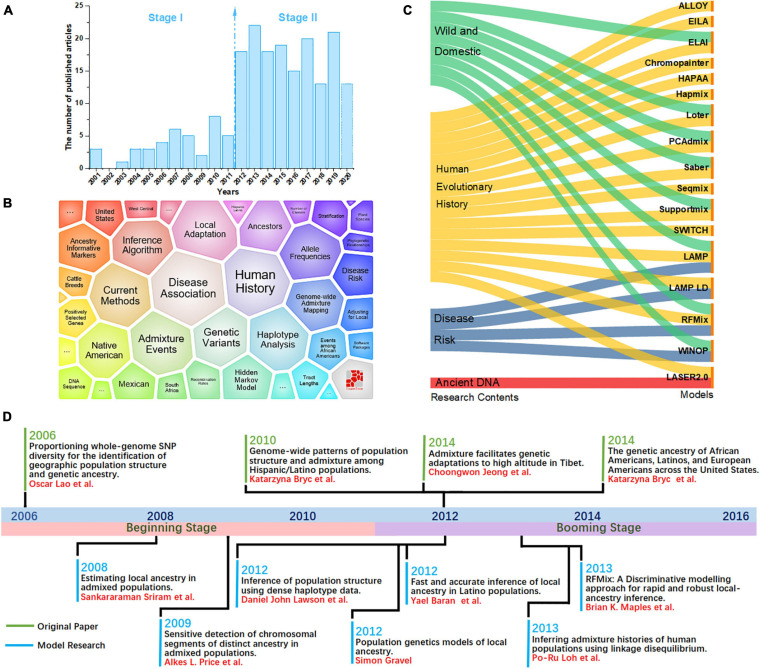
**(A)** Number of local ancestry inference (LAI) publications from 2000 to 2020. **(B)** A visual survey of the major topics on LAI by the Carrot II system. **(C)** Distribution of models across the top four research fields including wild domestication, history of human evolution, disease risk, and ancient DNA. **(D)** Timeline illustrating the development of LAI.

Here, we performed a bibliometric analysis of the LAI research. “Local ancestry inference” was selected as the search topic from 2000 to 2020 from the NCBI database.^1^ Each bibliographic record includes detailed information of published articles, including their titles, abstracts, and keywords. Figure 1A shows the number of published articles on the significant increase in LAI from 2012. Since 2000, when Chapman and Thompson (2001) published *Linkage Disequilibrium Mapping: The Role of Population History, Size, and Structure*, 186 articles have been published until 2020. The major topics in LAI research are shown in Figure 1B. The visual representation, known as a form tree, was generated using the clustering tool Carrot II (Cost et al., 2002) based on 40 clusters. The leading topics of research are disease association and human history. We analyzed the main contents of the cited articles for each model in Figure 1C, which illustrates that research on human history plays a leading role in LAI analysis and model development. Similarly, LAI research is also largely applied in disease risk, wildlife conservation, and domestication. Figure 1D shows four original types of research and seven model designs with top citations, which may play a driving role in the research of LAI. During 2006–2016, LAI research had been highly fascinating for various research groups; thus, LAI publications experienced a peak period. This research has gently and extensively infiltrated different fields of science and has kept on moving in the following years (Lao et al., 2006; Sankararaman et al., 2008b; Price et al., 2009; Bryc et al., 2010, 2015; Gravel, 2012; Lawson et al., 2012; Eaton and Ree, 2013; Loh et al., 2013; Maples et al., 2013; Moreno-Estrada et al., 2013; Jeong et al., 2014). To benchmark the computational efficiency and accuracy of the seven most used models (Chromopainter, LAMP, LAMP-LD, Loter, RFMix, Seqmix, and Supportmix), we simulated data using SLiM 3.2 (Messer, 2013) and estimated the average running time (ART), memory footprint size (MFZ), the mean squared error ($\text{MSE}=\frac{1}{n}\,\sum_{i=1}^{n}\left({\text{observed}}_{i}-{\text{predicted}}_{i}\right)$) for an individual genome, standard deviation (SD), and the coefficient of variation (CV) for each model. In the SLiM one, we initially generated two ancestor populations during 5,000 generations. The use of two initial populations differentiates into five admixed subpopulations with different infiltration rates after 4,000 generations. During the next step, differentiated individuals evolve freely during 5–1,000 generations, and every five generation is an interval. This step is repeated 20 times. Finally, we randomly selected 1,000 ancestral populations and 500 admixed populations to stimulate LAI in seven models. Table 2 shows further details regarding the simulation parameters and other simulation processes.

**TABLE 2:** Details for generating slim data.

	Slim1	Slim2	Slim3	Slim4	Slim5
Mutation rate	1e–8	1e–8	1e–8	1e–8	1e–8
Recombination rate	1e–6	1e–6	1e–6	1e–6	1e–6
Chromosome length	1e+6	1e+6	1e+6	1e+6	1e+6
Initial effective population size	2000	2000	2000	2000	2000
The number of generations producing true populations	5000	5000	5000	5000	5000
The number of ancestral populations	2	2	2	3	3
Effective ancestral population size	2000	2000	2000	2000	2000
Divergence time of ancestral population	4000	2000	200	2000	200
The number of admix populations	5	5	5	6	6
The number of selected ancestor individuals	1000	1000	1000	1000	1000
The number of selected admix individuals	500	500	500	500	500
Repetition times	20	20	20	20	20
Infiltration rate		AdmP1 = 0.1\0.9 AdmP2 = 0.2\0.8 AdmP3 = 0.3\0.7 AdmP4 = 0.4\0.6 AdmP5 = 0.5\0.5		AdmP1 = 0.1\0.1\0.8 AdmP2 = 0.2\0.1\0.7 AdmP3 = 0.3\0.1\0.6 AdmP4 = 0.4\0.1\0.5 AdmP5 = 0.5\0.2\0.1 AdmP6 = 0.6\0.2\0.2	
Generation number	5∼1000 (the interval is 5 generations)				

As shown in Table 3, we adopted seven models in SLiM 1–3 and six models in SLiM 4–5 because Seqmix can only handle two ancestral groups. The most efficient model is LAMP with respect to the run time (ART = 1.50 s) and memory size (MFZ = 53.74 Mb); however, its accuracy is slightly lower (1 – mean of MSE = 0.67) and the results are not stable (SD = 0.20). The primary reason is the total reliance of this model on biological parameters. Seqmix based on aligned bases turns out to be the most accurate (1 – mean of MSE = 0.86) and stable (SD = 0.08) model, while it is also efficient enough. Loter is the only model with a parameter-free process and general accuracy (1 – mean of MSE = 0.79) and fair stability (SD = 0.10); however, it requires a comparatively longer running time (ART = 2,506.70 s). The RFMix process has general accuracy (1 – mean of MSE = 0.80) and fair stability (SD = 0.10), but it consumes a lot of memory (MFZ = 2,472.29 Mb). A weighing between the pros and cons of the different models is shown in Table 4.

**TABLE 3:** Benchmark analysis of most used LAI models.

Model	ART/s	MFZ/Mb	1–Mean of MSE	SD	CV
Chromopainter	2243.56	309.20	0.73	0.18	0.24
LAMP	1.50	53.74	0.67	0.20	0.30
LAMP-LD	97.95	129.40	0.60	0.18	0.30
Loter	2506.70	269.84	0.79	0.10	0.13
RFMix	166.74	2472.29	0.80	0.10	0.13
Seqmix	31.11	1201.27	0.86	0.08	0.09
Supportmix	753.01	130.94	0.80	0.12	0.15

**TABLE 4:** Weighing of most used LAI models.

Model	Advantage	Disadvantage
Chromopainter	Moderate memory consumption and certain accuracy	Slower processing speed and unstable analysis results
LAMP	Fast processing speed and low memory consumption	Low accuracy and unstable analysis results
LAMP-LD	Fast processing speed and moderate memory consumption	Low accuracy and unstable analysis results
Loter	Moderate memory consumption, certain accuracy and stable analysis results	Slower processing speed
RFMix	Moderate processing speed, high accuracy and stable analysis results	High memory consumption
Seqmix	Fast processing speed, high accuracy and stable analysis results	High memory consumption
Supportmix	Moderate processing speed and memory consumption, high accuracy and certain stibility	–

**FIGURE 2:**
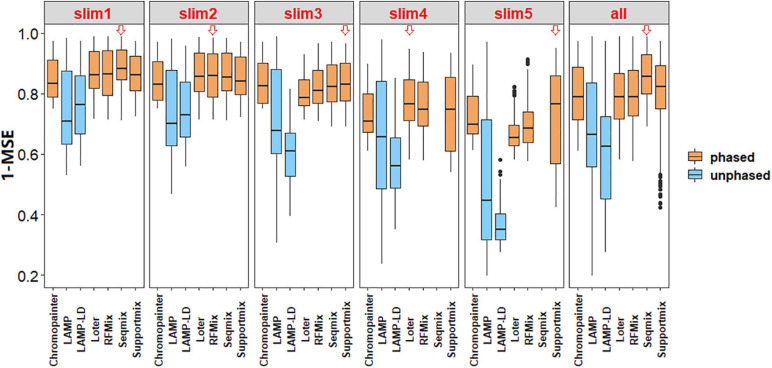
Box plots of the accuracy of local ancestry inference (LAI) using a benchmark. The *red hollow arrows* indicate a higher accuracy by the median comparison in this simulation. The results showed that phased data had higher accuracy in comparison with unphased data.

As shown in Figure 2, the phased data had a higher accuracy in comparison to the unphased data. Besides, there exists a significant difference between the phase and unphased results (1 – mean of MSE) in all the simulated values by each paired comparison in Tukey’s HSD (all *P* < 0.05). As shown in Table 3, the CV of the phased results is less than that of the unphased results in all simulated values, thus proving the higher stability of phased data.

## Discussion

### Current Situation and Existing Problems

Various challenges confront the researchers during inferring the local ancestry *via* genome-wide data. Firstly, several models need complex parameters, such as a genetic map and the number of generations since admixture, that are difficult to be supplied, particularly for non-model species. Secondly, some models only use haplotype information and unlinked markers are removed *via* the trimming step. With this process, many informative SNPs are lost. Thirdly, because some models exclude probable ancestral informative haplotypes, unmodeled LD could cause systematic biases in determining ancestry, which results in false-positive conclusions regarding the deviation in ancestry at specific loci. Lastly, ancestral segments are windows or blocks of varying lengths; however, existing models commonly use a window of fixed size for simplification. The total count of generations since admixture is inversely proportional to the length of ancestral segments. As the number of generations is hardly recognized, it is difficult to investigate the breakpoint or transition point for ancestral haplotypes based on the statistics of the ancestral group or even an individual’s genome.

### Model-Based Recommendation

We summarize these models with their distinct advantages and disadvantages as follows: (i) We recommend Seqmix if the genotype is hard to call, and aligned bases can be used directly in this model (Hu et al., 2013). (ii) ALLOY utilizes F-HMM and the haplotype structure of the compound state to improve its accuracy. We recommend this model if ancient and complex admixtures need to be analyzed (Rodriguez et al., 2013). (iii) We recommend Saber if high-density SNP panels exist; however, a potential weakness of M-HMM, compared with an HMM, is that when the genetic information on the ancestral populations is not rich, it will weaken the accuracy of the calculations (Tang et al., 2006). (iv) ELAI is appropriate for instances where researchers require detecting further structure of the haplotypes because of the two scales of LD in admixture and a two-layer HMM exists as independent upper-layer latent clusters that enforce structure on the haplotypes and other lower-layer latent clusters depicting ancestral haplotypes (Guan, 2014). (v) We recommend EILA if the researchers are interested in the estimation of recombination events. The model has the advantage of allowing the lack of ancestral populations’ high-quality haplotype information; however, a potential weakness of the *k*-means, unsupervised clustering, will weaken the stability of calculations (Yang et al., 2013). (vi) Loter uses DP to obtain a globally optimal solution, and its advantage is its being parameter-free (Dias-Alves et al., 2018).

### Integration With Other Methods

LAI incorporates other bioinformatics approaches and is widely used in different research fields, including breeding new varieties, protection of endangered animals and plants, and the prevention and treatment of human genetic diseases. In the study of population structure, the ADMIXTURE (Alexander et al., 2009) and STRUCTURE (Pritchard et al., 2000) models perform population allele frequencies and observe genotype probability by ancestry proportions. Both models can be used to assign global ancestry. They are applied in fine-matched corrected association research and are relatively consistent with the LAI results. Galaverni et al. better estimated the actual admixture proportions of the hybrids according to the combination of global and local ancestry inferences (Galaverni et al., 2017). About up to 50% of blocks of domesticated individuals were identified by PCADMIX in the hybrid genome. The results of the analysis were consistent with those estimated in ADMIXTURE at *K* = 2. In the study of domestication, the admixture compositions of select individuals with the minor allele for the peak markers of quantitative trait loci (QTL) were analyzed by LAI. For example, in one study, QTL were located in a chromosome segment substitution line (CSSL) population. This population comes from an interspecific cross between a wild aus-like *Oryza rufipogon* donor accession and cv. *Curinga* (an upland tropical japonica variety from Brazil). It was found that the CSSLs conferred a wild aus-like introgression across the target segment, which was beyond the rest of the CSSLs that carried the tropical japonica genotype (Wang et al., 2017). In the study of ancient DNA, the use of LAI and masking reconstruct population-specific surrogates of the ancestral components to yield entire genome. Yelmen et al. applied this technique to reconstruct population-specific surrogates of South Asian and West Eurasian populations, which complemented low-quantity and low-coverage availability and provided a substantial advantage (Yelmen et al., 2019).

### Application and Development

Wild populations significantly contribute to the adaptation of domesticated populations; therefore, their absence or presence is imperative for breeding and genetics-related studies. Many good traits exist in the wild population; however, they were lost during domestication. Some advantageous or disadvantageous alleles were located by constructing a hybrid population and were further assigned the corresponding ancestral source. This can help in understanding the molecular mechanisms behind the traits and in explaining the valuable pool of genetic resources found in wild populations. Domesticated rice (*Oryza sativa*) is adopted as an example. Some traits of wild rice (such as persistent seed dormancy and freely shattering seed) may have high adaptability if introgressed into weedy rice populations. Inversely, some traits of wild rice (prostrate plant architecture and sporadic seed production) are considered inappropriate for survival in domesticated rice. Given the potential combination of the advantageous and disadvantageous traits for weedy rice, it can be expected that introgression evidence of wild rice to weed rice would confer weed rice-adaptive traits to the specific genomic regions. Such as some regions were likely introgressed from wild accessions: *PROG1*, controlling prostrate *versus* erect growth; *qSW5*, controlling seed size; *sh4*, controlling grain shattering; *Bh4*, controlling hull color; *An-1*, controlling awn development; and *Rc*, controlling pericarp pigmentation (Vigueira et al., 2019). In another study, the analysis of wild caprids and whole genomes of domestic goats revealed ancient introgression evidence from a West Caucasian tur-like population to the ancestor of domestic goats. It was further revealed that the *MUC6* gene was an introgression locus with a strong selection signature and conferred enhanced immune resistance to gastrointestinal pathogens (Zheng et al., 2020). The third case is the wild yeast (*Saccharomyces eubayanus*). The lager-style beers are an interspecies hybrid (*S. eubayanus* × *Saccharomyces cerevisiae)*. It was found that the wild isolates of *S. eubayanus* are not the closest relatives of lager-brewing hybrids. Inversely, the genetic composition of lager yeasts was contributed by *S. eubayanus* strains with continuous variation, thus revealing the complex ancestries of lager yeasts (David et al., 2016). The LAI model can be a powerful tool for protecting wild species by identifying segments of the genomes of hybrids. In the research of Galaverni et al., domestic dogs (*Canis lupus familiaris*) can reproduce with wild wolves (*Canis lupus*), coyotes (*Canis latrans*), and golden jackals (*Canis aureus*). The gene pool of several wild canid populations were threatened by the widespread diffusion of stray dogs in human-dominated areas. Use of the LAI model and genotype–phenotype association procedures identified putative dog-derived causal mutations associated with phenotypic variants, thereby constituting a conservation strategy. Such as the black coat color, this trait is coded by a 3-bp deletion at the β-defensin gene *CDB103* that was possibly introduced into wolves by ancient hybridization with dogs (Galaverni et al., 2017).

The LAI model can be applied to the treatment and prevention of human genetic diseases by assigning ancestry to the chromosomal regions and applying admixture mapping to identify candidate genes. Dengue has become a worldwide health concern due to the increase in virus and vector dispersions. LAI analysis has proven that African ancestry has a protective effect against the dengue haemorrhagic phenotype in admixed Cuban population. This was further authenticated by identifying the corresponding candidate genes (Sierra et al., 2017). A similar study indicates that the Tibetans have a better altitude adaptation, on account of the introgression of associated haplotypes from Denisovans or Denisovan-related populations (Huerta-Sánchez et al., 2014). Besides, a recent example is that about 3,000 coronavirus disease 2019 (COVID-19) patients and control individuals were adopted, and it was found that a gene cluster can cause severe symptoms after SARS-CoV-2 infection. This genetic risk factor was caused by a genomic segment of a size of about 50 kb inherited from Neanderthals (Zeberg and Pääbo, 2020). Furthermore, this genomic segment was carried by about 50% South Asian and about 16% European people. In conclusion, these studies not only enhance our understanding of genetic diversity and natural history but also offer valuable evidence for the source of diversity among human beings, animals, plants, and model organisms.

## Author Contributions

JW and YZ wrote the paper. YL organized and designed the benchmark. YZ supervised the study and revised the manuscript. All authors have read and commented on the manuscript and approved the final version.

## Conflict of Interest

The authors declare that the research was conducted in the absence of any commercial or financial relationships that could be construed as a potential conflict of interest.

## Footnotes

1 https://pubmed.ncbi.nlm.nih.gov
